# Automated Disease Detection in Gastroscopy Videos Using Convolutional Neural Networks

**DOI:** 10.3389/fmed.2022.846024

**Published:** 2022-04-12

**Authors:** Chenxi Zhang, Zinan Xiong, Shuijiao Chen, Alex Ding, Yu Cao, Benyuan Liu, Xiaowei Liu

**Affiliations:** ^1^Department of Computer Science, University of Massachusetts Lowell, Lowell, MA, United States; ^2^Department of Gastroenterology, Xiangya Hospital of Central South University and Hunan International Computer Aided Diagnosis and Treatment for Digestive Disease, Changsha, China; ^3^Department of Computer Science, Brown University, Providence, RI, United States

**Keywords:** medical image, gastrointestinal disease, CNN, image classification, real-world video

## Abstract

A large percentage of the world's population is affected by gastric diseases ranging from erosion and ulcer to serious ailments such as gastric cancer, which is mainly caused by Helicobacter pylori(H.pylori) infection. While most erosions and ulcers are benign, severe cases of gastric diseases can still develop into cancer. Thus, early screening and treatment of all gastric diseases are of great importance. Upper gastroscopy is one such common screening procedure that visualizes the patient's upper digestive system by inserting a camera attached to a rubber tube down the patient's digestive tracts, but since the procedure requires manual inspection of the video feed, it is prone to human errors. To improve the sensitivity and specificity of gastroscopies, we applied deep learning methods to develop an automated gastric disease detection system that detects frames of the video feed showing signs of gastric diseases. To this end, we collected data from images in anonymous patient case reports and gastroscopy videos to train and evaluate a convolutional neural network (CNN), and we used sliding window to improve the stability of our model's video performance. Our CNN model achieved 84.92% sensitivity, 88.26% specificity, and 85.2% F1-score on the test set, as well as 97% true positive rate and 16.2% false positive rate on a separate video test set.

## 1. Introduction

There are a variety of gastric diseases that affect the health of a large fraction of the world's population, ranging from mild erosive gastritis to advanced cancer. A gastritis is characterized by the presence of map-like redness or diffuse redness in the stomach, with or without atrophy and the presence of erosion within the mucosal layer. Peptic ulcers are defects in the gastric or duodenal mucosa that extend through the muscularis mucosa. A gastric carcinoma with infiltration no deeper than submucosal layer can be considered early gastric cancer, while precancerous lesions include gastric ulcer, atrophic gastritis, intestinal metaplasia, and gastric polyps that may eventually develop into a gastric adenoma (1, 2). It was reported in Søgaard et al. (3) that the absolute 1–5 year risk of any GI cancer was 2.1% for patients with a gastric ulcer and 2.0% for patients with a duodenal ulcer. Other studies show that the malignancy rate in gastric ulcers spans a wide range between 2.4 and 21% (4, 5). Geographically, gastric cancer occurs at a very different rate in different parts of the world, with approximately half of all new cases occurring in East Asia (6). It is of great importance to develop a system that can detect gastric diseases, especially those with potential to become malignant, to help improve the screening and diagnosis of gastric cancer.

To reduce the potential for human error, a number of studies have been carried out to explore the use of machine learning to automatically classify gastrointestinal diseases. By replacing traditional feature engineering with convolutional neural networks (CNN) based deep learning models such as GoogleNet (7) and AlexNet (8), these studies achieved much better results on the task of classifying between images showing healthy tissues and images showing signs of various diseases. However, these models are often trained on images that are carefully selected during endoscopy procedures, with a stringent inclusion criteria that filters out blurry and low-contrast images, as well as images with visual obstructions such as bleeding and surgery instruments (9, 10). Furthermore, all current methods are either only evaluated at the image level or can only identify one or two specific types of diseases and are therefore unsuitable to be applied to the real-time video feeds in clinical settings.

Recently, there has been a large surge in applying deep learning to detect and classify gastric diseases, including ulcer, erosion, and cancer. Shichijo et al. (10) trained a 22-layer GoogleNet to classify helicobacter pylori infection. Takiyama et al. (9) used Single Shot Multibox Detector (SSD) (11) to detect gastric cancer, including images with standard white light, narrow band imaging, and chromoendoscopy using indigo carmine spraying, while excluding magnified or poor quality images. Khan et al. (12) proposed a deep learning based method to detect ulcer and classify several gastrointestinal diseases, using a modified mask regional CNN (Mask R-CNN) (13) model to segment detected ulcers. In classification, they fine-tuned a pretrained ResNet-101 through transfer learning and derived deep features from the training data, then supplying the features to a Multi-class Support Vector Machine (MSVM) for final classification. Luo et al. (14) developed a Gastrointestinal Artificial Intelligence Diagnostic Systems (GRAIDS), based on DeepLab-v3+ with one input and two outputs, to perform classification and segmentation on upper gastrointestinal cancer. Horie et al. (15) developed an esophagal cancer diagnosis system using Single Shot Multi-Box Detector. The closest work to our paper is done by Byrne et al. (16). The authors proposed a deep learning-based automated polyp detection system based on the Inception network architecture (17), which can achieve real-time assessment of colorectal polyps. The video segments used in the study are captured under the NBI mode by Olympus video recorders, and contain polyps of different sizes.

Despite the abundance of prior work, none of them except the last one can be used on the video level, either due to large models that cannot keep up with real-time video feeds or only being trained using clear images that are unsuitable for real-world videos, and all these works focus on image-level classification. The last work does achieve video-level inference, but it can only be used for NBI images.

**Figure 1:**
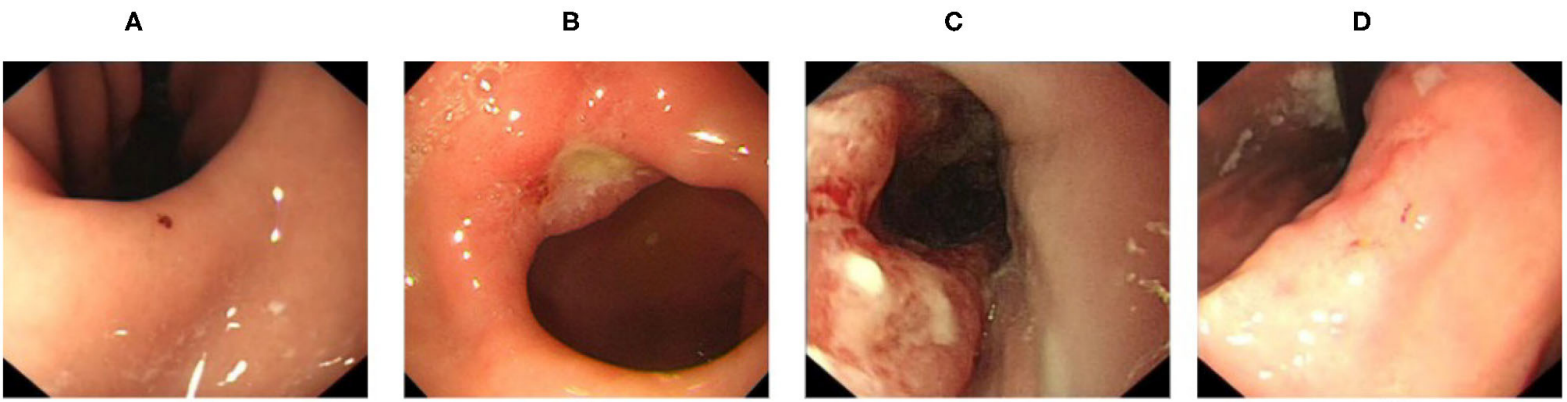
Disease category: **(A)** erosive, **(B)** ulcer, **(C)** early caner, and **(D)** advanced cancer.

In order to address these problems, we collected data of four types of diseases commonly screened for during upper endoscopy (erosion, ulcer, early cancer, and advanced cancer, visualized in Figure 1) from both patient reports and direct samples of endoscopy videos to train our models to handle the various complications arising from real-world videos. After that, we compared the performance of a variety of lightweight CNN models to distinguish gastric diseases from healthy images. We then built an gastroscopy video processing system to identify video segments containing signs of gastric diseases by combining our trained image-level model with a separately trained blurry image classifier to filter out blurry frames and the sliding window algorithm to improve stability. To the best of our knowledge, our method is the first effort which can distinguish aforementioned common gastric diseases from healthy images and be used in real-time.

Throughout our experiments, we also employed class activation mapping (CAM) (18), which highlights specific regions of an input image used by the trained models to generate their classifications, to verify that our model had actually learned meaningful features, instead of artifacts of the training data that are correlated with the image labels. This extra layer of sanity check allowed us to identify and correct several problems with our training data, which we document in this article for instructive purposes.

## 2. Materials

### 2.1. Data

We collected the data and generated accurate labels to train and evaluate our models, taking care to protect patient privacy by anonymizing all personal information. Our data is composed of two parts: one from 2,477 digital patient reports and one from real-world upper endoscopy videos.

#### 2.1.1. Data Collection From Patient Reports

From the electronic medical records (EMR) of the hospital, we extracted endoscopy images from patient reports between March 2015 and August 2020 using relevant keywords and saved them alongside the original reports, which contain detailed information about the operation, such as the date of the operation, equipment used, location of the sampled images, written description by the operating physician, lesion sizes and types, etc. All images extracted from a patient's record are displayed on a web page. Our collaborating endoscopists select the images for each specific disease, namely, erosion, ulcer, early cancer, and advanced cancer. The selected images are saved to our database with the corresponding labels, and are later used to train and test our model. Altogether, there are 1,798 erosive, 3,606 ulcer, 882 early cancer, 704 advanced cancer, and 4,805 healthy images. In our study, we group the different diseases into a single disease category, resulting in a total number of 6,990 disease images. For healthy class, we collect images from patient reports that do not have any disease label, creating a set of 4,805 images. The aggregation of the disease and healthy images is called dataset A.

#### 2.1.2. Data Collection From Endoscopy Videos

**Figure 2:**
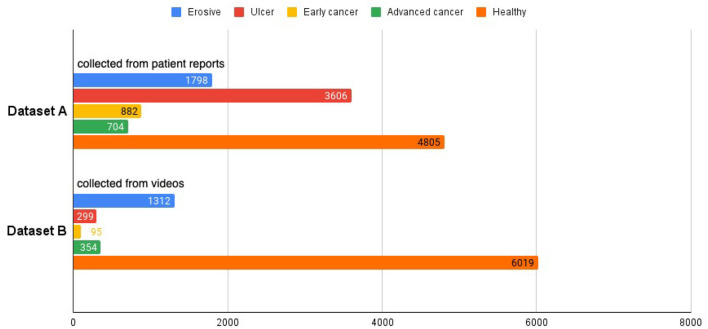
Dataset composition.

**Figure 3:**
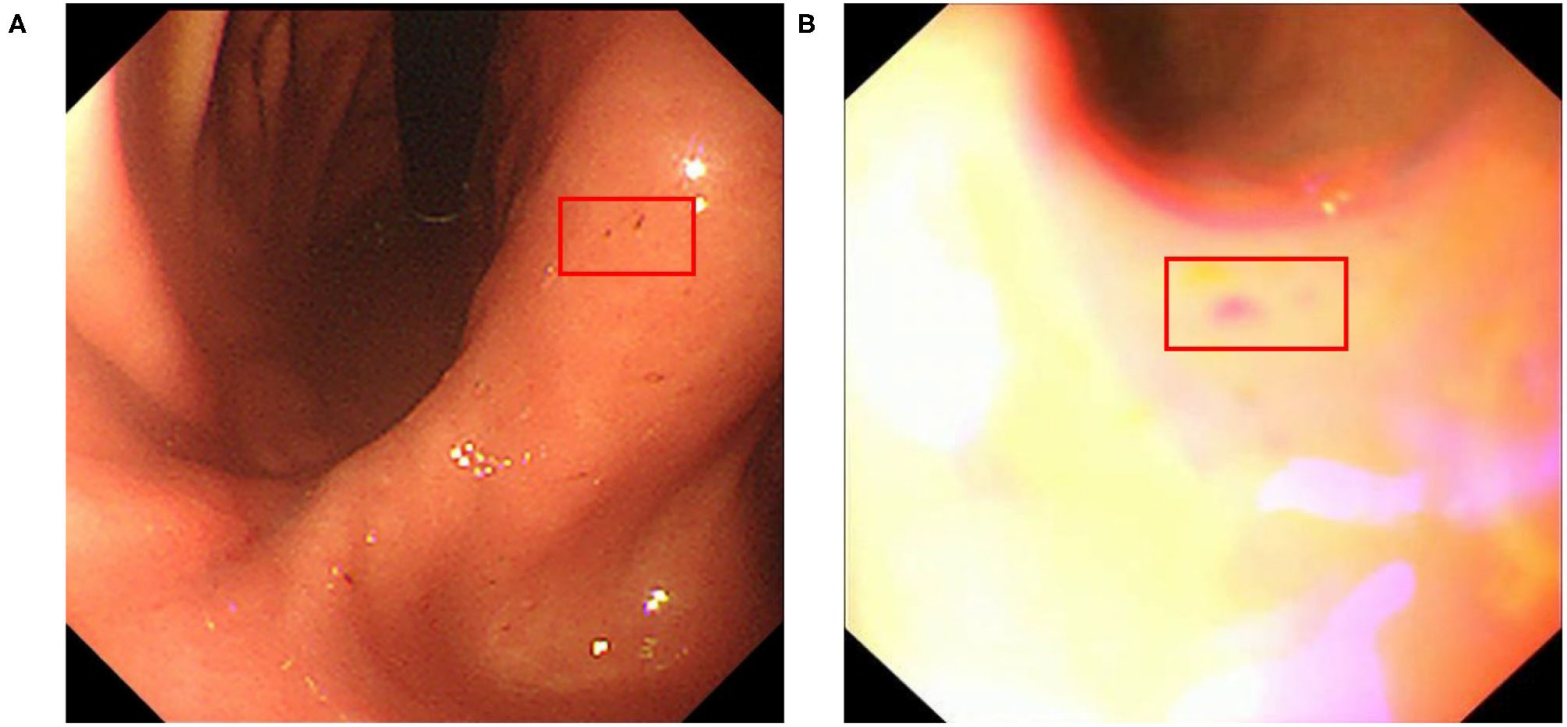
Example erosive images from datasets **(A,B)** red box indicates the location of erosive lesions.

We collected an additional 280 real-world upper endoscopy videos, also from Xiangya Hospital, between October 2019 and January 2020. We asked doctors from Xiangya Hospital to review each video, logging all time slots where the signs of diseases are within view of the camera along with the type of disease. Afterwards, we sample from the logged time slots for disease images and from untagged time slots for healthy images, yielding 2,060 disease images and 6,019 healthy images. Among these are 1,312 erosive, 299 ulcer, 95 early cancer, 354 advanced cancer, and 6,019 healthy images. These images form dataset B and supplement dataset A with important samples from real-world videos that greatly improve our classifier's clinical performance. An overview of the datasets can be seen at Figure 2, and we provide a side-by-side comparison between example images in dataset A and dataset B in Figure 3. As we can see, the image from dataset B is blurrier and has less contrast, which supplements dataset A's higher quality images to help our model deal with the real-world video images, which often suffer from a variety of artifacts such as motion blur and overexposure. We also collected a separate set of upper endoscopy videos and processed them similarly to evaluate our model's video level performance. We specifically asked the doctors to label 10 of these videos second by second to provide a better ground truth for video evaluation. In total, we have 3,110 erosive images, 3,905 ulcer images, 977 early cancer images, 1,058 advanced cancer images, and 10,824 healthy images.

#### 2.1.3. Sample Size Calculation

We use the following sample size formula (19) to calculate the number of positive samples and negative samples:


$$n=\frac{Z^{2}P\left(1-P\right)}{{\text{Δ}}^{2}},$$

where *Z* represents the *z*-score, *P* represents the expected prevalence, and Δ is the margin of error. We adopt the *z*-score as 1.96 (corresponding to 95% confidence interval), *P* value as 90%, and Δ as 0.01, to determine the required sample size as 3,458. In our training dataset, the numbers of positive and negative samples are 9,050 and 10,824, respectively, both larger than the required sample size.

## 3. Methods

**Figure 4:**
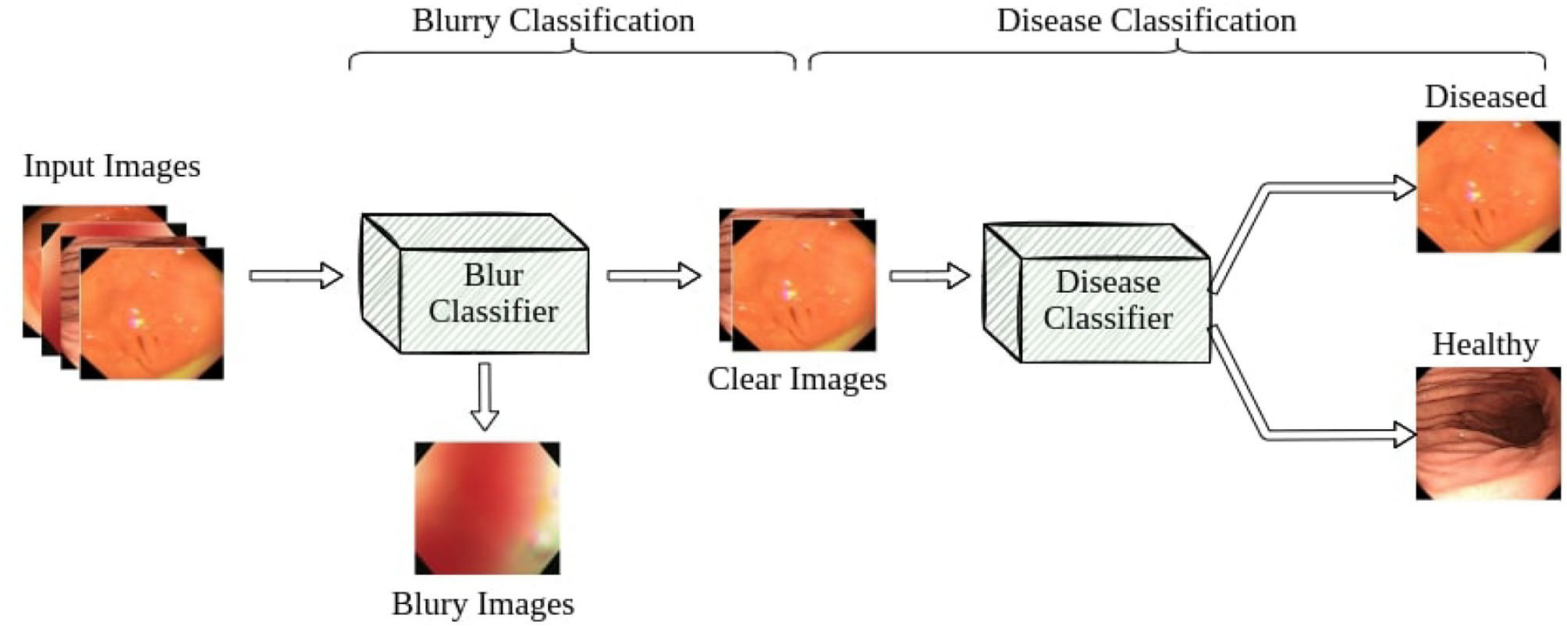
Overview of our gastroscopic disease classification system.

Our pipeline consists of two classifiers: a blur classifier that filters out uninformative blurry images and a binary disease classifier that detects frames with diseases. We then create a video processing system using these models to classify the video frame by frame, smoothing out the predictions using a sliding window. We present an illustration of the pipeline in Figure 4.

### 3.1. Blur Classifier

Blurry images are a major problem for endoscopy aid software in clinical settings. Existing applications that work well on clear test images usually struggle to accurately classify real-world video feeds, which are full of noise and blur. In our work, we design and attach a blur classifier to the front of our disease classifier to filter out blurry images and mitigate their impact. We manually selected 348 blurry images and 292 clear images to construct the dataset to cover most blurry scenes in real-world scenarios, including motion blur and out-of-focus blur caused by the camera moving or flushing of the intestines and stomach. We then trained our blur classifier using ResNet-50, achieving 94.7% accuracy on a test set composed of 229 images. We also increased the classification threshold to 0.7 to only remove very blurry frames and accept images with slight blurs for the next step.

### 3.2. Disease Classifier

The CNN-based classifier has dominated among computing vision areas during the past decade (20). From the original AlexNet to the most recent EfficientNet (21), the networks become more accurate and faster at the same time. For our diagnosis system, quick response and accuracy are both critical. In the compact network field, SqueezeNet (22) consumes a low amount of memory while maintaining the competitive performance. ResNet-50 and its variants also achieved fast inference speed among the ResNet family. For the more recent EffcientNet, the B4 version is most suitable for our task. We conduct an ablation study among those networks to find the best performing one for our study. Finally, we choose the ResNeXt-50 (23), a variant of Res50, as our classifier due to its highest accuracy on our datasets and short repose time.

We explored a variety of different lightweight classifier architectures for our disease classifier, including ResNet-50, ResNeXt-50, ResNet-101, SqueezeNet, and EfficientNet-B4. Based on their performance, we selected ResNeXt-50 for our video-level experiments. It is a variation of the classic ResNet, using a split-transform-merge strategy with a cardinality parameter to learn more abstract features with similar numbers of parameters. Its identical path design as ResNet also makes it easier to adopt and faster to inference from than InceptionNet.

### 3.3. Sliding Window

Random noise is inevitable in real-world videos. Although we have added a classifier to filter out blurry images, we cannot guarantee that all frames fed into the disease classifier are informative enough to make a correct prediction. The sliding window is a standard technique for limiting interference from outlier frames and reducing the false positive rate during video level inference. Instead of making decisions based on each individual frame, we consider the classifier's label of the past *k* frames, where *k* is the window size, and use a majority-vote mechanism to determine the actual inferred label. In experiments with more than two classes, if no particular label forms a majority at a given window, we default to the healthy class. A larger window size reduces false positive rate but also masks over more true positive cases, so we tune *k* to balance the two metrics.

## 4. Experiment

### 4.1. Evaluation

We evaluate the performance of our proposed approach using both standard image-level evaluation metrics and video-level metrics that we specifically designed for this purpose. The image-level metrics include precision, recall and F1 score, defined as follows:


(1) $$\begin{array}{c} \begin{array}{ccc} precision & = & \frac{TP}{TP+FP},recall=\frac{TP}{TP+FN},\\ F_{1} & = & 2\cdot \;\frac{precision\;\cdot \;recall}{precision\;+\;recall}, \end{array} \end{array}$$

where TP, FP, and FN stand for the number of true positive, false positive, and false negative outcomes, respectively.

Note that precision is the ratio of true positives to the total number of predicted positives, and recall is the number of true positives divided by the number of real positives in the dataset. Combining these two measures into a single metric, F1-score is the harmonic mean of the precision and recall, primarily used to compare the performance of two classifiers.

Our metric for evaluating video levels needs to reflect the performance of our system in real-life scenarios. It is critical for our system to identify disease instances as soon as possible after they appear on camera and send a warning to the physicians. With the help of this warning message, the physicians can avoid missing inconspicuous diseases and locate these disease areas more easily. However, it is still important to continue catching the disease while it is still visible. Additionally, for an optimal user experience, the system should send as few false positive notifications as possible. To capture these aspects of evaluation, we considered three different metrics for real-world video evaluation: true positive rate, coverage, and false positive rate.

The true positive rate (TPR) is calculated as follows. Within each disease period in the ground truth video, an alarm window is established at the beginning. If a positive prediction is made within the alarm window, we consider this as a hit, and the overall true positive rate is calculated as the ratio of hits to the number of ground truth disease periods. The size of the alarm window is a hyperparameter that we tune to evaluate how fast diseases can be detected by our model. For our experiments, we settle on an alarm window of 5 seconds.

Coverage rate (CR) is calculated by counting how many positive frames our model predicts during each ground truth disease period, and divide it by the number of frames within the disease period. The higher this rate, the fewer positive predictions our model misses.

The false positive rate (FPR) is the ratio of the number of positive predictions outside any ground truth disease period over the number of healthy frames in the video. This rate shows how many wrong positive predictions our model makes.

### 4.2. Model Training

We use both dataset A and B to train our disease classifier. We split dataset A into train and test sets case-wise to ensure that we do not have images from the same patient in both the train and test sets. For dataset B, we consider each video as one instance and split them instance-wise for the same reason. The train-test split ratio is 7:3. We use an Adam optimizer and a cosine scheduler to adjust the training, with an initial learning of 0.001 and a 10-epoch time cycle for the scheduler. Each model is trained for 60 epochs, and we select the one with the best F1 score to use for video evaluation.

## 5. Results

We explore a few different architectures for the disease classifier on dataset A+B, with results shown in Table 1. The performance of multi-class classification is presented in Table 2. Based on the overall performance, we choose ResNeXt50 for further video-level experiments. Table 3 presents the performance of our model trained on dataset A, dataset B, and datasets A+B without applying sliding window. We also test our system on ten finely-annotated videos, and report the result in Table 4. To reduce the false-positive rate, we investigate the effect of different sliding window sizes and thresholds, with results shown in Tables 5, 6.

**Table 1:** Image-level classification performance of different models on Dataset A+B.

	**Precision**	**Sensitivity**	**Specificity**	**F1 score**	**Kappa**	**MCC**
MobileNetV2	84.16%±0.3%	83.16%±0.4%	87.07%±0.3%	83.64%±0.5%	70.31%±0.8%	70.34 ± 0.2%
SqueezeNet	82.23%±0.8%	82.57%±1.1%	85.07%±0.3%	82.31%±0.3%	67.68%±0.4%	67.89 ± 0.4%
VGG16	84.19%±2.3%	85.24%±2.9%	86.67%±2.8%	84.60%±0.1%	71.83%±1.1%	71.95 ± 0.2%
GoogleNet	83.99%±1.4%	82.32%±0.5%	87.02%±0.4%	83.12%±0.6%	69.47%±1.1%	69.52 ± 0.2%
ResNet-50	85.59%±0.4%	84.01%±0.6%	88.28%±0.5%	84.72%±0.2%	72.40%±0.3%	72.47 ± 0.3%
ResNeXt-50	85.64%±1.2%	84.92%±1.8%	88.26%±1.4%	85.26%±0.6%	73.22%±1.1%	73.25 ± 1.1%
ResNet-101	85.25%±1.5%	84.26%±0.9%	87.92%±0.4%	84.69%±0.4%	72.26%±2.6%	72.34 ± 0.6%
EfficientNet-B4	86.69%±0.2%	84.69%±0.4%	89.21%±0.1%	85.66%±0.1%	74.09%±0.2%	74.13 ± 0.2%

**Table 2:** Precision, recall, F1-score of all multi-class classification experiments.

		**Healthy**	**Mild-disease**	**Severe-disease**	**Erosive**	**Ulcer**	**Early cancer**	**Advanced cancer**
{Healthy, mild-disease, severe-disease}	Precision Sensitivity Specificity F1-score	89.5% 86.9% 82.1% 88.2%	73.4% 76.9% 88.1% 75.1%	56.1% 57.1% 96.3% 56.6%	N/A	N/A	N/A	N/A
{Healthy, erosive, ulcer, early cancer, advanced cancer}	Precision Sensitivity Specificity F1-score	91.7% 85.2% 79.1% 88.3%	N/A	N/A	57.4% 67.4% 95.1% 62.1%	61.8% 70.6% 93.6% 65.9%	38.5% 45.5% 97.4% 41.7%	73.2% 52.7% 97.9% 61.3%

**Table 3:** Video-level performance using ResNeXt-50 trained on datasets A, B, and A+B, no sliding window, alarm window = 5 seconds.

	**TPR**	**CR**	**FPR**
Dataset A	97%	54.5%	27.2%
Dataset B	89%	34.3%	6.6%
Dataset A+B	97%	52.7%	16.2%

**Table 4:** ResNeXt-50 on fine-tuned annotated video.

	**TPR**	**CR**	**FPR**
Fine-annotated	92%	63%	14.1%
Original-annotated	87%	61%	18.8%

**Table 5:** Performance comparison with different sliding window sizes with a threshold of 0.5.

**Window size**	**TPR**	**FPR**
Size 20	89.4%	13.2%
Size 30	88.5%	12.8%
Size 40	87.7%	12.4%
Size 50	87.7%	12.1%

**Table 6:** Performance comparison with different thresholds with a window size of 20.

**Threshold**	**TPR**	**FPR**
0.5	89.4%	13.2%
0.6	88.6%	11.1%
0.7	87.7%	9.5%

### 5.1. Image Level Result

**Figure 5:**
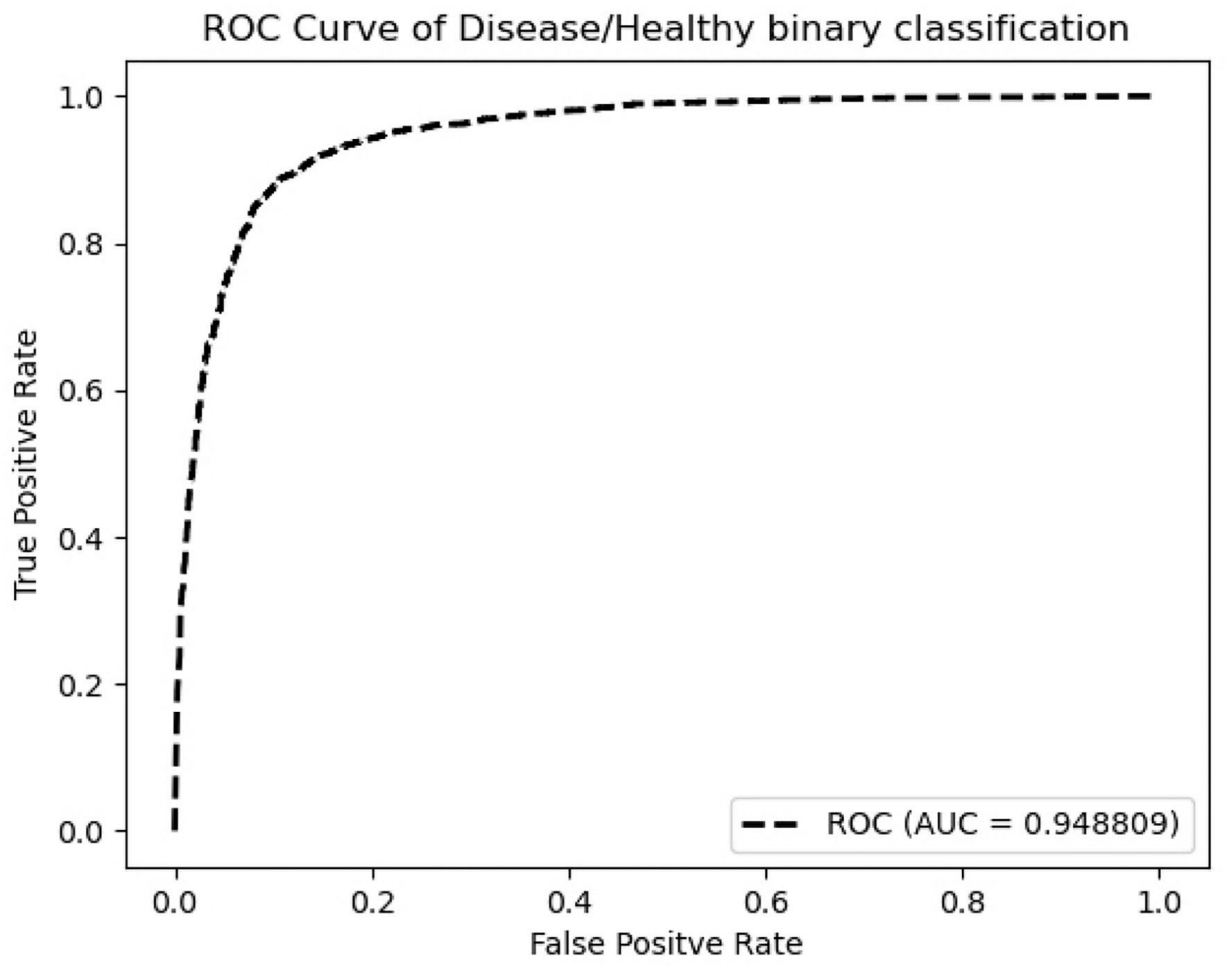
ROC curve of ResNeXt-50 on Dataset A+B.

According to Table 1, ResNeXt-50's F1 score is 85.26%±0.6%, which is superior to ResNet-50 because of the design of Cardinality Residual Block. Furthermore, ResNeXt-50 outperforms the lightweight SqueezeNet, MobileNetV2, conventional VGG16 models, while achieving comparable result with heavyweight models EfficientNet and ResNet-101. The sensitivity and specificity of ResNeXt-50 are 84.92±1.8%, 88.26±1.4%, respectively. In terms of the Kappa score and Matthews correlation coefficient, they all fall within the range of 61 to 80%, indicating that the prediction and the observation are highly concordant. Our further experiments are conducted using the ResNeXt-50 architecture, which provides the overall best performance. Figure 5 plots the ROC curve of the best performing ResNeXt-50 model, with an AUC score of 0.948.

**Figure 6:**
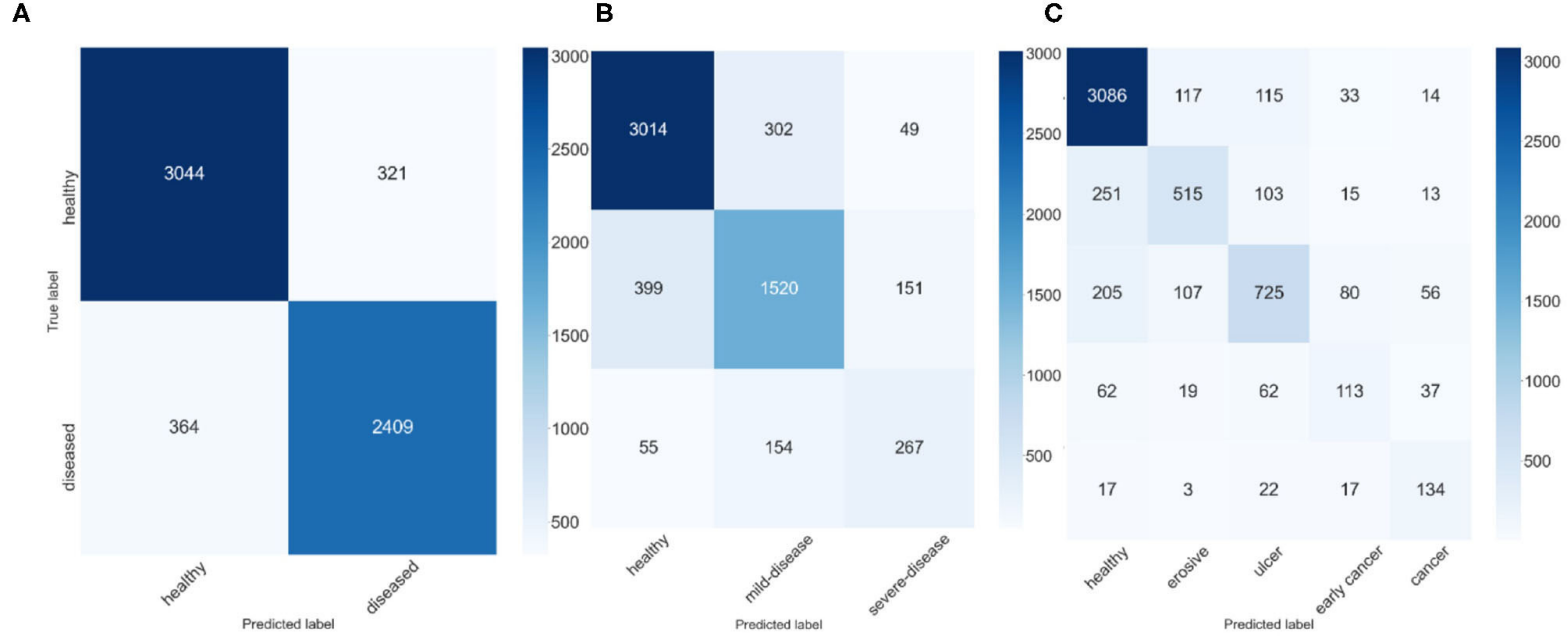
Confusion Matrices, **(A)** confusion matrix of 2-class experiment, **(B)** confusion matrix of 3-class experiment, and **(C)** confusion matrix of 5-class experiment.

**Figure 7:**
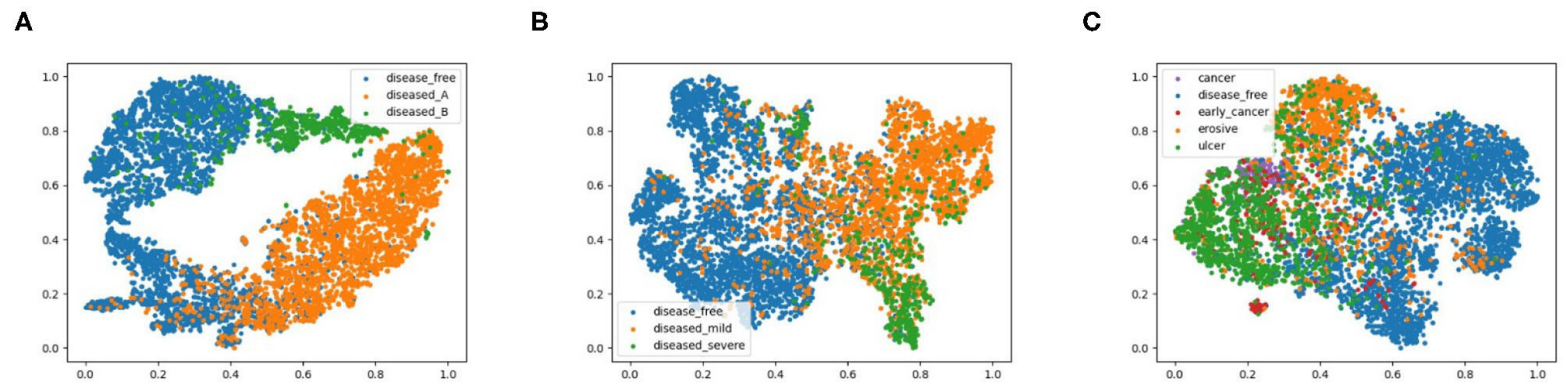
2D PCA analysis scatter for image classification experiments, **(A)** shows the 2-class experiments, and we also plot the disease from different data source with different colors, **(B,C)** represent the 3-class and 5-class experiments.

To explore the potential of our datasets, we also carry out additional multi-class (three-class and five-class) classification experiments. For the three-class experiments, we consider erosive and ulcer images as a mild disease class while early cancer and advanced cancer are combined into a severe disease class. For the five-class experiments, we treat each disease as a separate class. Table 2 shows the result of these multi-class experiments. The F1 scores for three- and five-class experiments are around 70 and 60%, respectively, which are much lower than the binary classification due to the similarity between different classes. For example, milder forms of early cancer have similar texture and color to ulcers; severe early cancer or even ulcer may look similar to advanced cancer; milder erosive and healthy images are also hard to distinguish. The confusion matrices and PCA analyses for these experiments presented in Figures 6, 7 further confirm the these observations.

### 5.2. Video Level Result

Performance on video is our primary concern, as this is the real application setting. We evaluate our models over 121 real gastroscopic operation videos, of which 46 of the them consist of labeled diseases. We calculated our three metrics—true positive rate, coverage, false positive rate—on models trained using dataset A, dataset B, and datasets A+B without applying sliding window, shown in Table 3. Although dataset A produces the highest TPR of 97%, its FPR is also the highest amongst the three. Without noisy images captured from actual videos, the model trained on dataset A is too sensitive with real-world videos. In contrast, the model trained on dataset B achieves 89% true positive rate. The model trained using datasets A+B balances the advantages of the two other models, lowering FPR to 16.2% while still maintaining a 97% TPR and 52.7% CR. Thus, we use the model trained on datasets A+B for all future experiments.

We further apply sliding windows to balance TPR and FPR. We explore a variety of window sizes and classification thresholds, which are the proportions of images in a given window that need to be predicted as disease for the frame to actually be considered a disease frame, in Tables 5, 6. As expected, increasing the threshold lowers both TPR and FPR, and increasing the window size lowers both TPR and FPR. To compromise between the two, we use a window size of 20 and a threshold of 0.5. With the sliding window, the TPR drops from 97 to 89.4%, the CR drops from 52.7 to 50.9%, and the FPR drops from 16.2% to 13.2%. We see a significant drop in TPR in exchange for a 3% decrease in FPR, which is more significant than one might think, the total denominator of FPR is the number of healthy frames, which is much larger than the number of disease frames.

The inference time of our system is about 19ms, and our experiments are ran on Intel i7 CPU and GTX 1080Ti GPU. The blur classifier takes about 10.6ms, The disease classify costs about 8.4ms, and the sliding window process time takes less than 1ms which can be ignored. The corresponding speed of our system is about 50 frame-per-second, which satisfies the real-time requirement of the system in gastroscopy.

**Figure 8:**
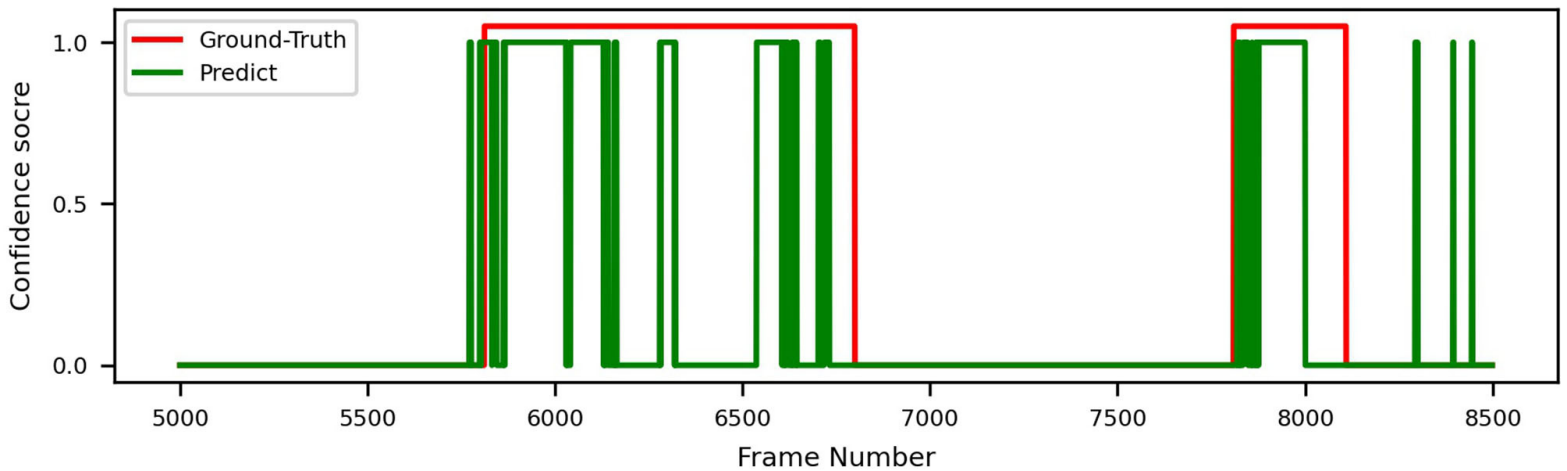
Video prediction and ground truth of ResNeXt-50, trained on datasets A+B, with sliding window.

In order to demonstrate intuitively how our models perform on real endoscopy videos, we plot the ground truth and our model's inference (with all techniques applied) in Figure 8. As we can see, the model effectively identifies each disease period with no false positives in the example.

## 6. Discussion

We apply several techniques to gain a deep understanding of our results. Figure 6 shows the confusion matrices of best ResNeXt-50 model in the experiments. For 3-class classification, we observe that there are much more incorrect predictions between “adjacent” classes (i.e., between healthy and mild-disease and between mild-disease and severe-disease) than between “faraway” classes (i.e., between healthy and severe-disease), indicating that while severe diseases (early cancer, cancer) share some similarities with mild diseases (erosive, ulcer), they look quite different from healthy ones. Healthy images are more likely to be mistaken for mild diseases than for severe diseases. Similar observations can be made for 5-class classification result.

We also conducted a principal component analysis (PCA) to visualize how the data are separated in two-dimensional space, shown in Figure 7. Specifically, we flatten the output features of the final convolutional layer in the model and select basis components with largest eigenvalues as the two dimensions, effectively projecting the feature vectors onto two dimensions. We labeled diseased images from dataset A and B with different colors to highlight the different feature representations from different data sources. We observe from Figure 7A that for 2-class classification, the diseased and healthy images are well separated in the 2-dimensional PCA plot, and the images from dataset A and B also fall in different regions. For 3-class and 5-class cases, images of different classes become more overlapped, consistent with their degrading performance as shown in Table 2. To further explore the results of different models, the repeated measures ANOVA test is performed over 50 samples. The results of the ANOVA test indicate that there is a significant between-model effect for the overall performance of the model, and there is a significant interaction of the model by the different results from performance metrics such as precision.

**Figure 9:**
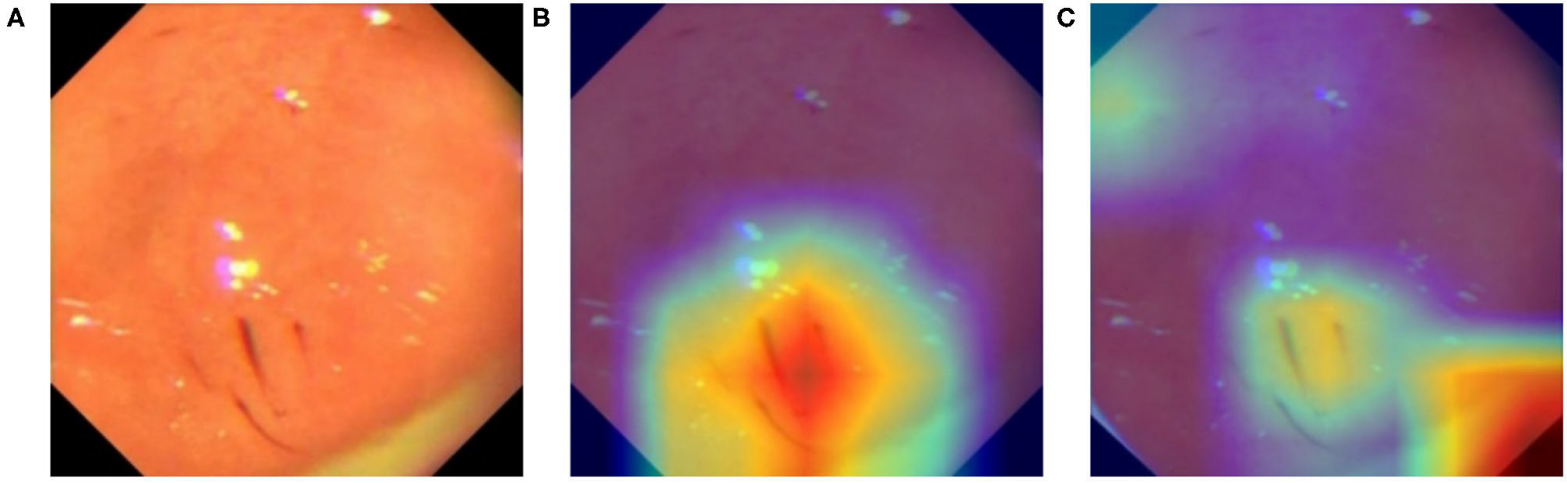
Activation map of an erosive image with GradCam. **(A)** Original image. **(B)** Heatmap (GradCam) on the corrected lesion location. **(C)** Flawed heatmap focused on irrelevant image corners.

For data from different equipment, the brightness, contrast, color, and even the border of the images exhibit distinct patterns. To ensure that our model is learning relevant features instead of induced artifacts to classify diseases, we apply GradCam (18) to generate heatmaps that highlight important regions of input images used by our model to generate its inferences. We choose the last layer of Layer-4 from ResNeXt-50 to create our activation maps, shown in Figure 9. From Figure 9C, we found our model overfitted to the black borders on the corners of the original image shown in Figure 9A, which have slightly different sizes depending on the equipment used to capture the image. This prompted us to modify the image preprocessing to additionally apply a uniform mask to ensure that each image has the same border dimensions. The corrected result is shown in Figure 9B, where the model now focuses on relevant features on the image.

During the evaluation of our system on the endoscopy video, we found three major issues with the disease section labeling. First, some parts of the videos exhibiting diseases were missed. Second, the timestamp of labeled disease periods are not very accurate, most of them deviating 1-3 seconds from the actual window. Finally, many labeled disease periods contain non-disease frames as the camera moves, which results in low coverage rates (CR). We selected 10 videos for our doctors to label at a finer granularity to improve the quality of ground truth, addressing the first two concerns. We ignored the third concern, since correctly identifying diseases is much more important than high coverage rate in our case. Table 4 shows that the model's TPR and FPR both improve with the finer annotation.

While our model performs well on both image and video levels in our experiments, there are still a few limitations that must be addressed in the future. Since our method is based on whole image classification, it is more sensitive to the inherent image characteristics that vary with different equipment than object detection or segmentation methods. Also, although we can show the approximate location of lesions using GradCam heatmap, the exact location of lesions cannot be determined as accurately as using object detection or segmentation methods. Moreover, since our current system focuses on distinguishing images of healthy and unhealthy conditions, it lacks the ability to discriminate between different gastric diseases. Additionally, like other deep learning approaches, the blackbox nature of CNN makes the model outputs difficult to explain. Last, but not least, considering our study was based on data from a single hospital, the generalizability of the findings needs to be confirmed in a larger study.

By utilizing our automatic gastric disease detection model, the endoscopists would be less likely to miss-diagnose precursor lesions for gastric cancer at an early stage, thus, reducing the economic burden associated with cancer treatment.

## 7. Conclusion

In this article, we designed and constructed an entire system for gastric disease classification, from early stage data collection to a final system that is evaluated against real-world videos. We explored a range of CNN architectures and studied the different performances on real-world videos with models trained from various data sources. Different sliding window filters were applied to balance the true positive and false positive rates. By combining clear images from patient reports and images sampled from real operation videos, our system is able to achieve 97% true positive rate, 52.7% coverage, and 16.2% false positive rate. By adding a sliding window, we can further reduce false positive rate to 9.5% while maintaining an 87.7% true positive rate.

As our dataset is labeled with rich class annotations that distinguish between different diseases, we can further optimize multi-class classification on top of binary classification in the future. We can also add instance level annotations to our dataset and train object segmentation models like Faster-RCNN (24) and SSD (25), which would allow our system to generate more precise highlighting of lesions and cancers for physicians.

## Data Availability Statement

The original contributions presented in the study are included in the article/supplementary material, further inquiries can be directed to the corresponding author/s.

## Ethics Statement

The studies involving human participants were reviewed and approved by Ethic Committee of the Xiangya Hospital of Central South University. Written informed consent for participation was not required for this study in accordance with the national legislation and the institutional requirements.

## Author Contributions

CZ and ZX have an equal contribution to method design and implementation. YC and BL provide algorithm advice for the system. AD contributes to data collection. SC and XL provide medical-related advice for the system. All authors contributed to the article and approved the submitted version.

## Conflict of Interest

The authors declare that the research was conducted in the absence of any commercial or financial relationships that could be construed as a potential conflict of interest.

## Publisher's Note

All claims expressed in this article are solely those of the authors and do not necessarily represent those of their affiliated organizations, or those of the publisher, the editors and the reviewers. Any product that may be evaluated in this article, or claim that may be made by its manufacturer, is not guaranteed or endorsed by the publisher.

## References

[1] Waldum H Fossmark R. Gastritis, gastric polyps and gastric cancer. Int J Mol Sci. (2021) 22:6548. 10.3390/ijms22126548 34207192 PMC8234857

[2] Hu B El Hajj N Sittler S Lammert N Barnes R Meloni-Ehrig A. Gastric cancer: classification, histology and application of molecular pathology. J Gastrointest Oncol. (2012) 3:251. 10.3978/j.issn.2078-6891.2012.021 22943016 PMC3418539

[3] Søgaard KK Farkas DK Pedersen L Lund JL Thomsen RW Sørensen HT. Long-term risk of gastrointestinal cancers in persons with gastric or duodenal ulcers. Cancer Med. (2016) 5:1341–51. 10.1002/cam4.680 26923747 PMC4924392

[4] Klang E Barash Y Levartovsky A Lederer NB Lahat A. Differentiation between malignant and benign endoscopic images of gastric ulcers using deep learning. Clin Exp Gastroenterol. (2021) 14:155. 10.2147/CEG.S292857 33981151 PMC8107004

[5] JM MPV FJ JP . Analysis of the clinical benefits and cost-effectiveness of performing a systematic second-look gastroscopy in benign gastric ulcer. Gastroenterol y hepatol. (2008) 32:2–8. 10.1016/j.gastrohep.2008.07.002 19174093

[6] Kim B Cho SJ. Endoscopic screening and surveillance for gastric cancer. Gastrointest Endosc Clin. (2021) 31:489–501. 10.1016/j.giec.2021.03.004 34053635

[7] Szegedy C Liu W Jia Y Sermanet P Reed S Anguelov D . Going deeper with convolutions. In: Computer Vision and Pattern Recognition (CVPR). Boston, MA (2015).

[8] Krizhevsky A Sutskever I Hinton GE. ImageNet classification with deep convolutional neural networks. In: Pereira F, Burges CJC, Bottou L, Weinberger KQ, editors. Advances in Neural Information Processing Systems. vol. 25. Red Hook, NY: Curran Associates, Inc. (2012).

[9] Hirasawa T Aoyama K Tanimoto T Ishihara S Shichijo S Ozawa T . Application of artificial intelligence using a convolutional neural network for detecting gastric cancer in endoscopic images. Gastric Cancer. (2018) 21:653–60. 10.1007/s10120-018-0793-2 29335825

[10] Shichijo S Nomura S Aoyama K Nishikawa Y Miura M Shinagawa T . Application of convolutional neural networks in the diagnosis of Helicobacter pylori infection based on endoscopic images. EBioMedicine. (2017) 25:106–11. 10.1016/j.ebiom.2017 29056541 PMC5704071

[11] Liu W Anguelov D Erhan D Szegedy C Reed S Fu CY . SSD: single shot multibox detector. In: Lecture Notes in Computer Science. Cham: Springer (2016). p. 21–37.

[12] Khan MA Khan MA Ahmed F Mittal M Goyal LM Hemanth DJ . Gastrointestinal diseases segmentation and classification based on duo-deep architectures. Pattern Recognit Lett. (2020) 131:193–204. 10.1016/j.patrec.2019.12.024

[13] He K Gkioxari G Dollár P Girshick R. Mask R-CNN. In: 2017 IEEE International Conference on Computer Vision (ICCV). Venice (2017). p. 2980–8.

[14] Luo H Xu G Li C He L Luo L Wang Z . Real-time artificial intelligence for detection of upper gastrointestinal cancer by endoscopy: a multicentre, case-control, diagnostic study. Lancet Oncol. (2019) 10:20. 10.1016/S1470-2045(19)30637-0 31591062

[15] Horie Y Yoshio T Aoyama K Yoshimizu S Horiuchi Y Ishiyama A . The diagnostic outcomes of esophageal cancer by artificial intelligence using convolutional neural networks. Gastrointest Endoscopy. (2018) 08:89. 10.1016/j.gie.2018.07.037 30120958

[16] Byrne MF Chapados N Soudan F Oertel C Pérez ML Kelly R . Real-time differentiation of adenomatous and hyperplastic diminutive colorectal polyps during analysis of unaltered videos of standard colonoscopy using a deep learning model. Gut. (2019) 68:94–100. 10.1136/gutjnl-2017-314547 29066576 PMC6839831

[17] Szegedy C Liu W Jia Y Sermanet P Reed S Anguelov D . Going deeper with convolutions. In: Proceedings of the IEEE Conference on Computer Vision and Pattern Recognition. Boston, MA (2015). p. 1–9.

[18] Selvaraju RR Cogswell M Das A Vedantam R Parikh D Batra D. Grad-CAM: visual explanations from deep networks via gradient-based localization. Int J Comput Vis. (2019) 128:336–59. 10.1007/s11263-019-01228-7

[19] Pourhoseingholi MA Vahedi M Rahimzadeh M. Sample size calculation in medical studies. Gastroenterol Hepatol Bed Bench. (2013) 6:14. https://www.ncbi.nlm.nih.gov/pmc/articles/PMC4017493/24834239 PMC4017493

[20] Schmidhuber J. Deep learning in neural networks: an overview. Neural Netw. (2015) 61:85–117. 10.1016/j.neunet.2014.09.003 25462637

[21] Tan M Le Q. EfficientNet: rethinking model scaling for convolutional neural networks In: Chaudhuri K, Salakhutdinov R, editors Proceedings of the 36th International Conference on Machine Learning. vol. 97 of Proceedings of Machine Learning Research. Long Beach, CA: PMLR (2019). p. 6105–14.

[22] Iandola FN Han S Moskewicz MW Ashraf K Dally WJ Keutzer K. SqueezeNet: AlexNet-level accuracy with 50x fewer parameters and <0.5MB model size. arxiv:1602.07360Comment: In ICLR Format. (2016).

[23] Xie S Girshick R Dollár P Tu Z He K. Aggregated residual transformations for deep neural networks. (2017).31141794

[24] Ren S He K Girshick R Sun J. Faster R-CNN: towards real-time object detection with region proposal networks. IEEE Trans Pattern Anal Mach Intell. (2017) 39:1137–49. 10.1109/tpami.2016.2577031 27295650

[25] Liu W Anguelov D Erhan D Szegedy C Reed SE Fu C . SSD: single shot multibox detector. CoRR. (2015) abs/1512.02325.

